# Apoptosis Induced by Piroxicam plus Cisplatin Combined Treatment Is Triggered by p21 in Mesothelioma

**DOI:** 10.1371/journal.pone.0023569

**Published:** 2011-08-17

**Authors:** Alfonso Baldi, Maria Teresa Piccolo, Maria Rosaria Boccellino, Aldo Donizetti, Irene Cardillo, Raffaele La Porta, Lucio Quagliuolo, Enrico P. Spugnini, Francesca Cordero, Gennaro Citro, Massimo Menegozzo, Raffaele A. Calogero, Stefania Crispi

**Affiliations:** 1 Department of Biochemistry, Section of Pathology, Second University of Naples, Naples, Italy; 2 Gene Expression & Human Molecular Genetics Laboratory, Institute of Genetics and Biophysics, CNR, Naples, Italy; 3 S.A.F.U. Department, Regina Elena Cancer Institute, Rome, Italy; 4 Campania Regional Operating Center (COR) of the National Mesothelioma Registry (ReNaM) - Department of Experimental Medicine, Second University of Naples, Naples, Italy; 5 Bioinformatics and Genomics Unit, Department of Clinical and Biological Science, University of Turin, Turin, Italy; Institut Jacques Monod, France

## Abstract

**Background:**

Malignant mesothelioma (MM) is a rare, highly aggressive tumor, associated to asbestos exposure. To date no chemotherapy regimen for MM has proven to be definitively curative, and new therapies for MM treatment need to be developed. We have previously shown *in vivo* that piroxicam/cisplatin combined treatment in MM, specifically acts on cell cycle regulation triggering apoptosis, with survival increase.

**Methodology/Principal Findings:**

We analyzed, at molecular level, the apoptotic increase caused by piroxicam/cisplatin treatment in MM cell lines. By means of genome wide analyses, we analyzed transcriptional gene deregulation both after the single piroxicam or cisplatin and the combined treatment. Here we show that apoptotic increase following combined treatment is mediated by p21, since apoptotic increase in piroxicam/cisplatin combined treatment is abolished upon p21 silencing.

**Conclusions/Significance:**

Piroxicam/cisplatin combined treatment determines an apoptosis increase in MM cells, which is dependent on the p21 expression. The results provided suggest that piroxicam/cisplatin combination might be tested in clinical settings in tumor specimens that express p21.

## Introduction

Malignant mesothelioma (MM) is a rare, highly aggressive tumor, accounting for less than 1% of all cancer deaths in the world [1], that arises from the surface of serosal cells of the pleura, peritoneum, and pericardium. The association between exposure to asbestos and MM development is commonly accepted. Epidemiological data indicate that in the next 30 years this disease will cause a quarter of a million of deaths in Europe in individuals exposed to asbestos [2]. The prognosis is generally poor, with a reported median survival from presentation ranging from 9 to 12 months in either untreated or treated patients [3].

Treatment of MM patients has included supportive therapy, surgery, chemotherapy and radiotherapy [4]. Overall, clinical benefits of conventional therapies are marginal, with chemotherapy as the choice treatment, taking into account that surgery and radiotherapy have limited benefits in highly selected patients - reaching a median survival of approximately 1 year. To date no chemotherapy regimen for MM has proven to be curative, and new therapies for MM treatment are being developed testing different drug combinations, that might be used as new therapies, or as part of new combined multi-modality treatments, with sequential surgery and/or radiotherapy.

The advent of genome-wide analyses that greatly enhanced the comprehension of the molecular changes, cancer-type distinctive, has allowed to shift cancer therapies from broad-spectrum treatments towards cancer-specific and molecular-targeted treatments, showing efficacy and a limited toxicity to normal cells. Furthermore, analysis of the pathways specifically de-regulated in cancer, have led to develop specific tumor inhibitors, as the farnesyltransferase inhibitor [5], the anti-VEGF (vascular endothelial growth factor) antibody bevacizumab [6], or the proteasome inhibitor bortezomib [7]. Similar drugs have been tested also in MM, as well as in the pre-clinical study based on cisplatin and bortezomib, reporting enhanced apoptosis and increased cisplatin cytotoxicity [8]. Among the combined chemotherapy regimens for MM, two proved to be favourable to palliation: pemetrexed plus cisplatin [9] and gemcitabine plus cisplatin [10].

A different combined treatment recently described by our group in MM used the non steroidal anti-inflammatory drugs (NSAIDs) piroxicam combined to cisplatin. This drug combination showed an anti-tumor effect, with increasing survival both *in vitro* and *in vivo*, as demonstrated in a murine orthotopic model of MM [11].

NSAIDs are commonly used as anti-inflammatory and analgesic drugs. They are non selective inhibitors of both cyclooxygenase-1 (COX-1), an enzyme constitutively expressed in many tissues, and cyclooxygenase-2 (COX-2), that is expressed at very low levels in most tissues [12]. COX-2 can be induced by cytokines and stress in various tissues and it is overexpressed in many cancers. The first studies associating NSAIDs treatment with a reduced cancer risk, were performed on colon cancer [13]. Since then, the antineoplastic effects of NSAIDs have been evaluated in many randomized clinical trials [14] [15] and on several *in vitro* and *in vivo* experimental MM models. In particular, NS398 produced a significant reduction of proliferation level in MM cell lines, [16] while celecoxib resulted efficient in inhibiting mesothelioma cell growth [17].

In a previous work we have demonstrated a significant anti-proliferative effect of piroxicam (P) in two mesothelioma cell lines not expressing COX-2, MSTO-211H and NCI-H2452, treating them with piroxicam alone or in combination with cisplatin (C). Drugs combination resulted in a synergistic effect, suggesting that piroxicam might sensitize MM cells to cisplatin cytotoxicity acting via a COX-independent mechanism. The results were confirmed *in vivo*, in a mouse MM model indicating that piroxicam and cisplatin association specifically acts on cell cycle regulation triggering apoptosis, and may hold promise in the treatment of MM [11]. Finally in spontaneous MM in pets, we recently have been able to show that piroxicam/cisplatin combination has remarkable efficacy at controlling the malignant effusion secondary to MM in our samples [18].

Starting from this background, the goal of this work was to dissect, at a molecular level, the effects of this combined treatment. Molecular changes responsible for the anti-tumor effect following the combined treatment were initially investigated by whole genome transcription profling. Specifically, we used Affymetrix microarray technology to identify differentially expressed genes in MSTO-211H cell lines after the piroxicam/cisplatin combined treatment. We associated apoptosis activation of the combined treatment to p21 expression, since apoptosis enhancement is impared upon silencing of p21. These results suggest a novel mechanism for this drug combination that might be tested also in other human cancers.

## Results

### Piroxicam and cisplatin combined treatment induces apoptosis in MSTO-211H cells

Previous studies from our laboratory established a role in mediating cell proliferation for the piroxicam/cisplatin combined treatment. We showed that piroxicam acts on MM cells reducing proliferation levels in a dose-dependent manner. Furthermore, as revealed by our group, in a MM ortothopic model, mice treated with combined therapy showed a prolonged survival and a tumor growth reduction. We assumed that piroxicam could exert its effects via COX-independent mechanisms because MSTO-211H cells express at very low levels COX-2 proteins [11].

**Figure 1:**
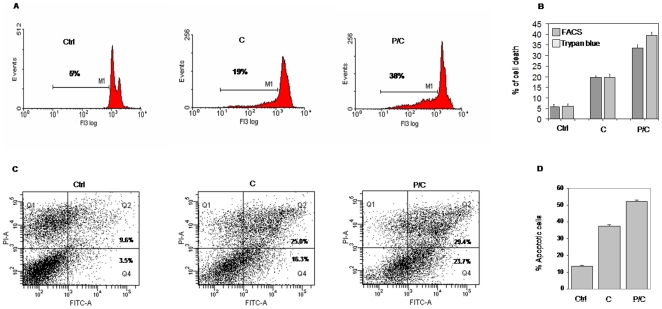
Piroxicam and cisplatin combined treatment induces apoptosis. A, MSTO-211H cells were exposed to cisplatin or to piroxicam and cisplatin and DNA content was analyzed by flow cytometry analysis. After 24 hours untreated (Ctrl) and treated cells (C, P/C) were collected, labeled with propidium iodide (PI) and analyzed. B, Cell viability analysis with the trypan blue. C, Apoptosis analysis by Annexin V-FITC/PI (Q1: necrosis; Q2: late apoptosis; Q3: healthy cells; Q4: early apoptosis). D, data summary of the apototic index. Data were performed on three independent experiments with comparable results. Ctrl: cells, C: cisplatin, P/C: piroxicam and cisplatin.

To further elucidate the effect of combined treatment on cell cycle regulation and the downstream signalling, we exposed MSTO-211H cells to both cisplatin and piroxicam/cisplatin in a time course experiment, using the drug concentration able to reduce cell proliferation by 50%, as we have previously showed [11]. Apoptosis was investigated by means of DNA distribution in flow cytometry analysis, using untreated cells as control. After single cisplatin treatment, we detected a 14% of apoptotic induction, while the comparison of cell DNA content between piroxicam/cisplatin and untreated cells, revealed a 33% of apoptosis increase after 24 hours treatment compared to control (Figure 1A). This analysis revealed no apoptotic induction at 8 hours both in single or in combined treatment (data not shown). These results were confirmed measuring the cell viability using the trypan blue method (Figure 1B). Apoptosis was further evaluated with Annexin V-FITC/PI staining confirming that combined treatment induced up to 37% apoptosis increase compared to control (Figure 1C).

**Figure 2:**
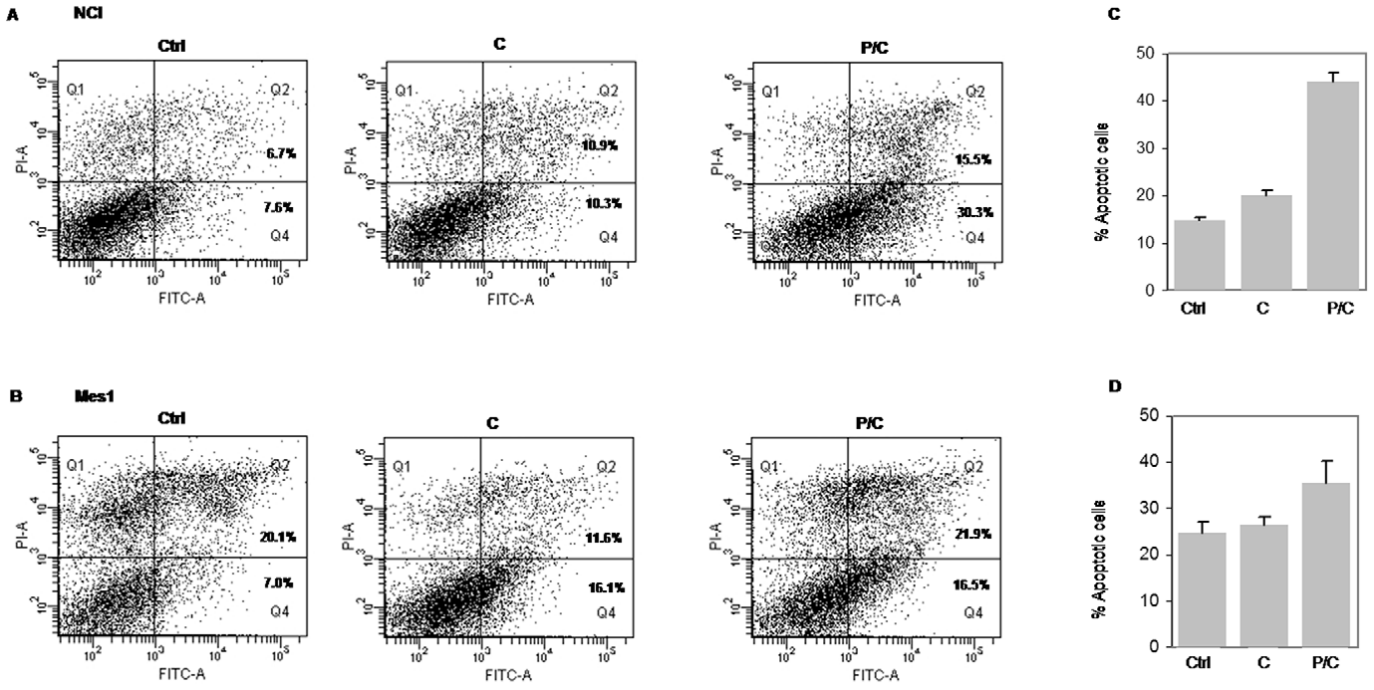
Apoptosis induction after combined treatment in MM cell lines. A and B, Apoptosis analysis by Annexin V-FITC/PI in NCI (A) and Mes1 (B) (Q1: necrosis; Q2: late apoptosis; Q3: healthy cells; Q4: early apoptosis). C and D, data summary of the apototic index. Cells were treated as described above for the MSTO-211H. Data were performed on three independent experiments with comparable results. Ctrl: cells, C: cisplatin, P/C: piroxicam and cisplatin.

To analyze if the effect exerted by piroxicam and cisplatin could be viewed as a general characteristic of MM cells, we analyzed apoptosis induction following the combined drug treatment in other MM cell lines. In particular NCI, Mes1 and Mes2 were treated as described above, then apoptosis was evaluated with AnnexinV-FITC/PI. NCI and Mes1 cell lines showed a similar apoptotic increase after combined treatment (Figure 2). We were unable to detect any significant apoptotic event in Mes2 cells upon single or combined treatment (data not shown).

### Genome-wide profiling analysis leads to identify genes involved in apoptosis enhancement following combined treatment

In order to analyze, at a molecular level, the effect of the combined treatment, and to identify the relative pattern modifications, we performed a transcriptional profiling on HGU133A arrays, using MSTO-211H cells treated with piroxicam, cisplatin or with piroxicam and cisplatin. Differential expressed genes in treated cells were detected comparing their expression respect to untreated cells.

On the basis of the above reported apoptotic induction, drug treatments were done at times in which apoptosis induction was undetectable (8 h) or present (24 h). Biological triplicates were generated for each prototypic situation and data were analyzed using the oneChannelGUI Bioconductor package [19].

The complexity of the data set was reduced removing the non-significant probe sets, resulting in a total of 4,247 out of the 22,283 probe sets present in the microarray. To assess differential expression, we used an empirical Bayes method [20] together with a false discovery rate correction of the P-value [21]. Specifically, genes were selected using a corrected p-value≤0.05 and |log2(fc)|≥1. We detected a total of 536 differentially expressed probe sets (Table S1).

**Figure 3:**
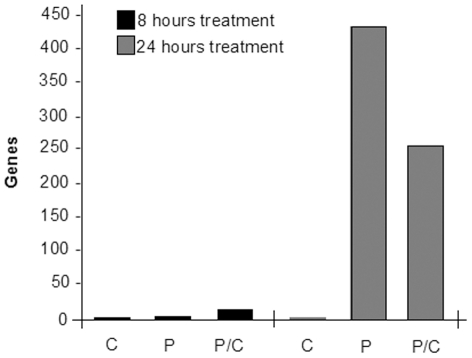
Differentially expressed genes enriched after single or combined treatment. Gene numbers after different time and drug treatments are shown. It is evident that only a 24 hour treatment reveals a consistent number of genes both in single piroxicam treatment and in the combined one. C: cisplatin; P: piroxicam; P/C: piroxicam and cisplatin.

To analyze in detail deregulated genes, and to identify a direct correlation to apoptosis induction, we performed a functional analysis using “Ingenuity Pathways Analysis” (IPA7.0, Ingenuity System®). As shown in Figure 3, we observed a consistent number of differentially expressed genes only after 24 h treatments both in piroxicam and in piroxicam/cisplatin. We were unable to detect differentially expressed genes upon cisplatin treatment, thus supporting the hypothesis that the cisplatin-induced cytotoxicity might be enhanced by piroxicam through the modulation of specific endogenous effectors as for the previously described HtrA1 – a serine protease that acts as a tumor suppressor-like protein [22]. Genes deregulated in the combined treatment were further analyzed in IPA for their molecular and cellular function and functional network. The analysis identified *Cancer*, *Cell Cycle and Cellular Growth and Proliferation* as the top three categories among the known affected biological function (Table 1) and *Cell cycle, Cellular movement and Cancer* as the most representative functional network.

**Table 1:** Top molecular and cellular functions at 24 hours piroxicam/cisplatin treatment.

Name	P- value	Molecules
Cancer	1.15E-13–9.71E-03	143
Cell cycle	4.14E-13–9.53E-03	125
Cellular growth and proliferation	4.28E-10–9.71E-03	94
Cellular movement	4.74E-09–8.96E-03	28
Cell death	5.61E-08–9.71E-03	146

To find out the mechanism underlying the enhanced apoptosis sensitivity in the combined treatment, we then focused our attention to genes associated to the above mentioned functional network. The network includes many cell cycle regulators; most of them with an opposite fold change in the single piroxicam treatment (see Table S1). Among them, we found CDKN1A (p21) one of the few genes up-regulated in this network (Table 2).

**Table 2:** Genes associated to Cell Cycle, Cellular Movement, Cancer Functional Network.

Associated Genes	Fold change
ASPM	−1.4
BCCIP	−2.04
BIRC5	−1.39
BUB1	−1.36
BUB1B	2.48
CCNA2	−2.71
CCNB1	−1.17
CCNB2	−1.07
CDC2	−1.20
CDCA3	−1.29
CDK2	−1.01
CDKN1A	3.32
CDKN3	−1.27
CEP55	−1.08
DDB2	−1.5
DLG7	−1.23
ECT2	−1.35
FEN1	1.89
FOXM1	−1.54
KIF14	−2.73
KIF20A	1.08
KIF23	−1.43
KIF4A	1.02
LGALS3BP	−1.72
MCM4	1.17
NCAPD3	1.20
NDC80	−1.07
PBK	−1.24
PHGDH	−1.76
PRC1	−1.17
RACGAP1	−1.2
TTK	−2.09

**Figure 4:**
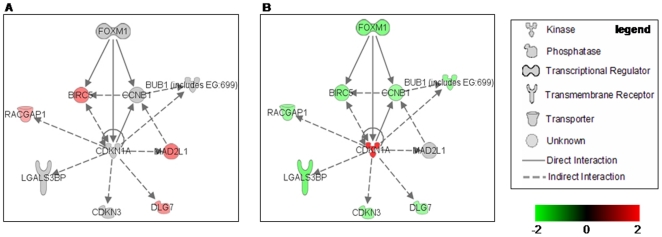
IPA functional pathway analysis. p21 functional relationship with other differentially expressed genes involved in cell cycle progression detected in this work. A, Expression after 24 hours treatments with piroxicam. B, Expression after 24 hours treatments with piroxicam/cisplatin. For each gene the relationship and the expression (red up-regulated, green down-regulated) are shown. Arrows indicate the direction of the relationship.

To better analyze the p21 function we used IPA to find functional relationship with other genes involved in cell cycle progression that could account for the apoptosis increase detected in the combined treatment. As shown in Figure 4B, p21 is connected to various genes, most of them down-regulated in the combined treatment.

Microarray results were confirmed by quantitative real-time reverse-transcription polymerase chain reaction (qRT-PCR). The analysis was performed on MSTO-211H cells for all the genes depicted in Figure 4 both after single piroxicam or combined piroxicam/cisplatin treatment. We also tested their expression, on samples previously described by our group [23], where microarray analysis was used to compare human MM samples with respect normal pleura to detect MM associated genes. As reported in Table 3, qRT-PCR data were in good agreement to the microarray results, as the array expression values were confirmed for almost all genes either in cells or in human samples. The results obtained were in agreement with other published works (Table 3) and they also reinforced the idea that p21 might be an important effector of the combined treatment.

**Table 3:** Validation of Array-Based Gene Expression Profile by Real-Time PCR in MM cell after P or P/C treatment and in human MM samples.

	MSTO11Ha)			Mesotheliomab)		
Gene name	Fold Change		Real time validation	Fold Change	Real time validation	Association to Mesothelioma
	P	P/C				
BIRC5	1.50	−1.39	Yes	3.59	Yes	[47], [48], [49]
BUB1B	-	−1.36	No	3.88	No	
CCNB1	-	−1.17	Yes	3.44	Yes	[47], [50]
CDKN1A	-	3.32	Yes	−0.61	Yes	[51]
CDKN3	-	−1.27	Yes	2.44	Yes	
DLG7	1.27	−1.23	Yes	3.89	Yes	
FOXM1	-	−1.54	Yes	-	-	[50]
LGALS3BP	-	−1.72	Yes	0.74	Yes	
MAD2L1	1.53	-	No	3.55	Yes	
RACGAP	1.01	−1.22	No	2.12	No	

a) after 24 hours treatment.

b) human sample [18].

### p21 protein profiling following combined treatment

**Figure 5:**
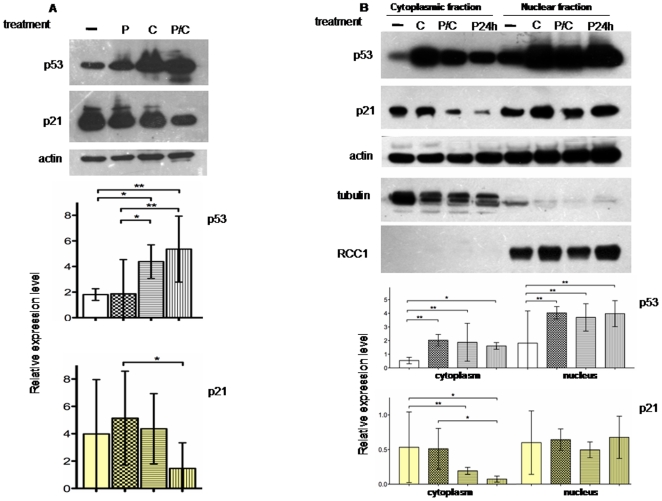
p21 protein is differently expressed in sub- cellular compartment. A, Western blot analysis and relative expression level on p53 and p21 proteins after 24 hours P, C or P/C treatment in MSTO11H. The analysis reveals an increase of p53 levels after C treatment probably related to the cisplatin-induced cellular stress. Indeed p21 levels appear decreased in the P/C combined treatment. Total proteins were incubated with p21 antibody, or p53 antibody. B, Western blot analysis and relative expression level on p53 and p21 proteins in cytoplasmic and nuclear subcellular fractions. Most of the p53 protein is localized in the nucleus and there is a similar result for p21. In addition the p21 nucleus/cytoplasm ratio increases in the prolonged piroxicam pre-treatment before adding cisplatin (lanes P24h). Proteins were probed with specific cytoplasmic (tubulin) or nuclear (RCC1) antibodies to exclude fractions cross-contamination. In all the experiments, actin was used as loading control. Histograms of relative expression level refer to p53 and p21 normalized expression and derived by the analysis of three independent experiments. Statistical analysis was done as indicated in Material and Methods. -: untreated cells, P: piroxicam; C: cisplatin; P/C: piroxicam and cisplatin P24h: piroxicam and cisplatin after piroxicam pretreatment.

p21 was initially identified as a p53-target gene, a tumor suppressor activated in response to DNA damage [24]. Because our microarray analyses did not detect any transcription deregulation of p53 expression, we wondered if we could detect, between single and combined treatments, a p53 differential expression at protein level. We performed a Western blot analysis in MSTO-211H using total protein extracts. As shown in Figure 5A, we detected an increase of p53 levels in cisplatin treatment, probably related to the cisplatin-induced cellular stress that acts through nuclear DNA binding [25], as well as in piroxicam/cisplatin treatment. Western blot analyses could not detect p21 protein increase and, in agreement with previously reported data [26] we noticed a decrease in the P/C treatment (Figure 5A).

To refine our knowledge on p21 expression at protein level we also investigated its subcellular localization. We analyzed protein expression either in cytoplasm or in nuclear extracts. As shown in Figure 5B, an increase in nuclear localization for p53 was found, as a consequence of cisplatin-induced cellular stress [25]. We also observed a similar effect for p21 which was mainly localized in the nucleus. Furthermore, we observed that p21 nucleus/cytoplasm ratio increased to a greater extent when we prolonged the piroxicam treatment for additional 24 hours before adding cisplatin (Figure 5B, lanes P24h) p21 nuclei shifting in the P/C combined treatment well agree with the observed apoptosis increase according to recent studies that address a dual role for p21 [27]. It has been reported that p21 can regulate cell cycle progression through inactivation of the cyclin-dependent kinase (Cdk)/cyclin complexes that are localized in the nucleus when active, and that the enhancement of p21 is linked to reduced expression of CDK and to cell growth inhibition. Despite this p21 inhibitory function, the inhibition of CDK activity determines the inactivation of the retinoblastoma tumor suppressor protein (pRB) that in turn sequesters E2F1 (E2 Family Member 1), thus leading to apoptosis induction [28].

### p21 silencing prevents apoptosis after piroxicam/cisplatin combined treatment

Before performing further investigation on p21 we sequenced in MSTO-211H cells all p21 coding exons, confirming the absence of any mutation. To gain insight the functional role of p21 in apoptosis observed after the P/C combined treatment, we silenced p21 expression by means of small interfering RNA technology (siRNA) and analyzed the effects on the cell viability after drug treatments.

**Figure 6:**
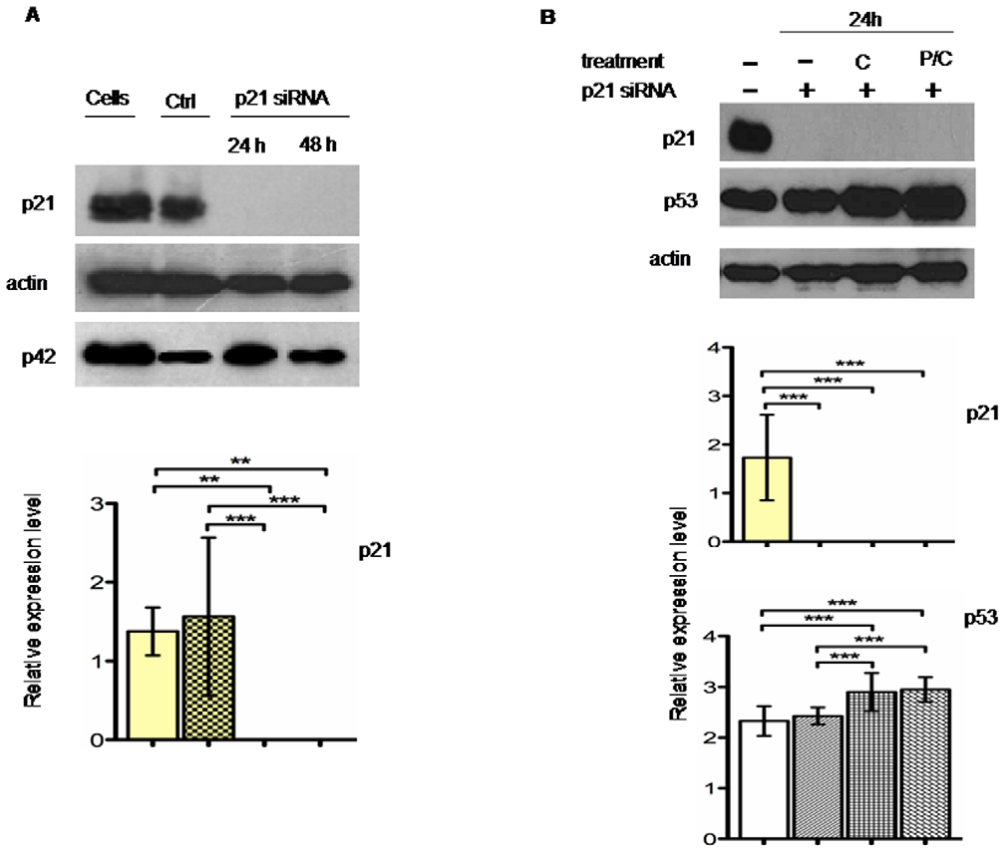
Effects of p21silencing on protein expression. A, Western blot analysis and relative expression level on p21 expression after p21 siRNA transient experiment. MSTO cells transfected with control or p21 siRNA were harvested at 24 or 48 hours after transfection. Total proteins were incubated with p21 antibody, or p42 antibody as internal control. Complete silencing was detected both at 24 and 48 hours. B, Western blot analysis and relative expression level on p53 expression after 24 hours, C or P/C combined treatment of MSTO-211H p21 silenced cells. 24 hours after silencing, cells were exposed to different drugs as indicated before protein extraction. Total proteins were incubated with p21 antibody, or p53 antibody. In all the experiments, actin was used as loading control. Histograms of relative expression level refer to p53 and p21 normalized expression and derived by the analysis of three independent experiments. Statistical analysis was done as indicated in Material and Methods. Cells: untreated cells; Ctrl: cells transfected with control siRNA; C: cisplatin; P/C: piroxicam and cisplatin.

Silencing was confirmed analyzing p21 protein levels. As shown in Figure 6, the protein was completely absent in p21 siRNA- transfected cells both at 24 or 48 hours after transfection, even in presence of drug treatments (Figure 6B).

**Figure 7:**
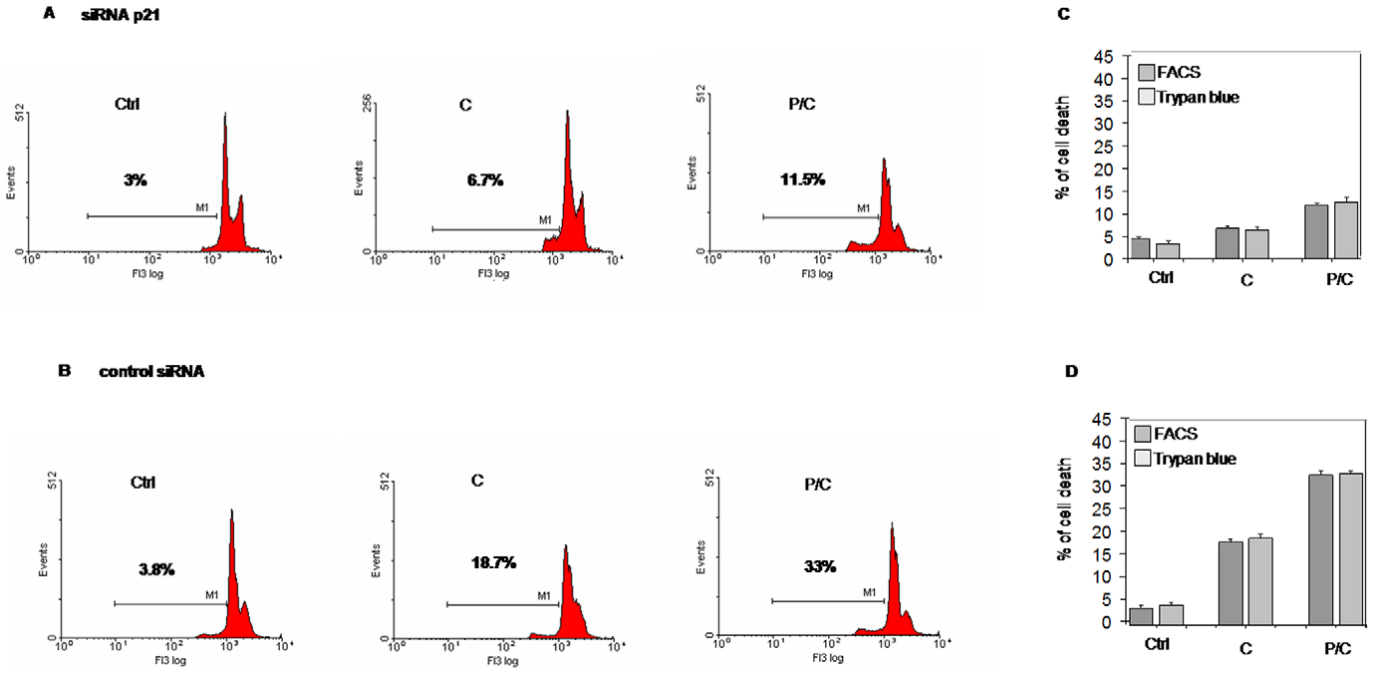
Apoptosis decrease in p21 silenced cells after piroxicam/cisplatin treatment. MSTO-211H cells were exposed to cisplatin or to piroxicam and cisplatin after 24 hours transfection with p21 siRNA or with the control siRNA. After transfection cells were drug-treated for additional 24 hours, then DNA content was analyzed by flow cytometry analysis with propidium iodide staining treating cells. A, p21 silenced cells show a marked apoptosis reduction after the combined treatment. B, Apoptosis was completely restored in the same experiments using control siRNA. C and D, Cell viability analysis with the trypan blue showed a reduced apoptosis only in p21 silenced cells. Data were performed on three independent experiments with comparable results. Ctrl: cells, C:cisplatin, P/C: piroxicam and cisplatin.

To analyze the p21 silencing effects on cell cycle, we measured the DNA content by flow cytometry analysis after silencing. Analyses were carried out on cells exposed to cisplatin or to piroxicam/cisplatin 24 hours after transfection. Figure 7 shows that upon p21 silencing, cisplatin single treatment induced apoptosis activation comparable with untreated cells, while we observed a marked decrease in the percentage (70%) of apoptotic cells in combined treatment (Figure 7A). Apoptosis was instead unaffected using a control siRNA (Figure 7B). These results were confirmed measuring the cell viability using the trypan blue method (Figure 7 C, D).

The above mentioned observations, demonstrate a tight relationship between p21 and apoptosis. If we also take in account that, under the same conditions, p53 protein level is not affected (Figure 6B), we can conclude that apoptosis induced by the combined treatment is mediated by p21 in p53 – independent way.

In this view we have verified the presence of a direct correlation among p21 silencing and some of its downstream genes linked to cell cycle effects (Table 3), also detected by the microarray analysis.

Microarray analyses revealed that the majority of transcription changes was detected after 24 hours treatment with piroxicam or with piroxicam/cisplatin and that the functional classes most affected by these changes are associated to cancer, cell cycle, cellular growth and proliferation. Specifically we observed that p21-related genes are all down-regulated in combined treatment, and that they are also characterized by opposite expression trend when compared to piroxicam alone (Table S1).

These genes have a role in cell growth and mitosis and they are essential for mitotic progression. Furthermore, most of them are considered cancer therapeutic targets.

Specifically, BIRC5, a member of the inhibitor of apoptosis (IAP) gene family, has been shown to inhibit apoptosis and enhance proliferation [29]. BIRC5 is up-regulated in almost all human tumors and its functional involvement, in apoptosis as well as in proliferation, leads to consider it as a new target for cancer treatment [30].

Furthermore, BUB1 and MAD2L1 are required for spindle checkpoint functions and for right metaphase chromosomal alignment [31]. BUB1 is important in recruiting other spindle checkpoints at the centromere and it is involved in tumor cell proliferation because its suppression determines apoptotic cell death. MAD2L1 in association with the cyclin B-ubiquitin ligase, is part of the anaphase-promoting complex, controlling the metaphase-anaphase transition. Depletion of these mitotic control proteins is associated to premature senescence and this phenotype is triggered by p21 [32].

Galectin-3-binding protein (LGALS3BP) – belongs to a protein family with high affinity for beta-galactoside and it is expressed in many tumor cells being associated to carcinogenesis. Interestingly, breast carcinoma cells overexpressing LGALS3BP, show apoptosis resistance in response to anticancer treatment [33].

We also found down-regulated two genes involved in citokinesis: RACGAP1 and DLG7. RACGAP1 is a Rho GTPase that forms the central spindlin complex, a complex essential for the assembly of a microtubule structure and for the subsequent formation of the contractile ring that, in turn, drives cytokinesis [34]. DLG7 is an essential component of the mitotic apparatus required for the assembly of the bipolar spindle that has oncogenic activity because it promotes cell survival. DLG7 is tightly regulated along the cell cycle - with increasing transcription levels from G_1_/S to G_2_/M - and its depletion determines chromosome congression delay [35]. It has been described as overexpressed in human hepatocarcinoma [36] and MM [23].

FOXM1 is instead a transcription factor required for mitosis progression whose loss determines spindle defects and centrosome amplification [37]. According to previously reported data, we found FOXM1 down-regulation linked to reduced expression of two direct transcriptional targets: CCNB1 - a key regulator of the G_2_/M checkpoint of the cell cycle, and CDKN3 - a gene required for the G_1_/S progression, whose expression results down-regulated in absence of FOXM1 [38].

Particularly interesting are the results obtained on CDKN3. CDKN3 expression is completely modified upon p21 silencing, resulting in an up-regulation both at RNA and protein levels (Figure S1). It was recently shown that CDKN3 expression is inversely correlated to p21 induction and that CDKN3 down-regulation negatively affects cell growth [39].

## Discussion

Evasion from apoptosis is one of the fundamental hallmarks of cancer, and apoptosis resistance is one of the major mechanisms related to drug resistance in tumour cells. Recent studies have showed that combined therapies acting on cell cycle - through pro-apoptotic proteins or specific miRNA - enhance tumor sensitivity to drugs [40].

Here we report that piroxicam/cisplatin combined treatment exerts an apoptotic effect on MM cells. Genome-wide transcriptome analyses led us to identify p21 as the possible apoptosis mediator acting as downstream target of the piroxicam/cisplatin treatment.

p21 belongs to the CDK (cycline-dependent kinase) family inhibitors that act on kinase activity of the CDK-cyclin complexes. p21 acts as a regulator of cell cycle progression at G1, inhibiting the activity of cyclin-CDK2 or -CDK4 complexes required for G1/S transition [41].

As a proliferation inhibitor, p21 plays an important role in preventing tumor development. Ectopic overexpression of p21 leads to cell growth arrest in G1 and G2 and this arrest is accompanied by phenotypic markers of senescence in the cell [32]. p21 promotes apoptosis through repression of different genes involved in cell cycle progression. Microarray data and qPCR provided the basis for the hypothesis that p21 plays a key role in piroxicam functionality in the view of a sensitization of the cells to cisplatin treatment. However the presence of discrepancy between transcription and translation level of p21 in the combined treatment highlighted the need of further investigations to understand the role of p21.

Specifically the presence of differential expression at transcriptional level of p21 upon the P/C combined treatment prompted us to hypothesize a role of p21 in the effects induced by the combined treatment. Although silencing of p21 impairs the functionality of the P/C combined treatment, reinforcing the idea of an involvement of p21 in the mechanism of action of P/C treatment, p21 transcription changes are not translated at protein level. However, we have observed that p21 localization changes upon the combined treatment, resulting in a nuclear accumulation of p21.

Recent studies provide evidences on the functional role of p21 in function of its cellular localization. Specifically it has been shown that p21 in its nuclear localization is associated to anti-proliferative functions as instead p21 cytoplasmic localization is linked to cell cycle progression and to anti-apoptotic functions [27].

Therefore, the increase in nuclei localization of p21 observed here upon the P/C combined treatment (Figure 5B) well agree with the above mentioned published data and provide new incite on the mechanism of action of the P/C combined treatment.

Interestingly, we have also observed in MM patients a significant positive relationship between p21 transcription expression level and their overall survival [42]. Therefore, determination of p21 expression might bear a prognostic significance in patients affected with MM.

In conclusion, the results shown here in combination with our previous data [11], lead us to suggest that piroxicam/cisplatin treatment of MSTO-211H cell line determines *in vivo* a tumor regression and a survival increase which is dependent by p21.

## Materials and Methods

### Cell lines and reagents

The human mesothelioma cell lines MSTO-211H, NCI-H2452 (NCI), IST-Mes1 (Mes1) and IST-Mes2 (Mes2) were obtained from the American Type Culture Collection (Rockville, MD, USA). IST-Mes1 (Mes1) and IST-Mes2 (Mes2) were obtained from the ISTGE (Istituto Nazionale per la Ricerca sul Cancro – Genova). Piroxicam (Pfizer, New York, NY) was a 60-mmol/L injectable solution; cisplatin (Pharmacia-Italia, MI, Italy) was a 50 mmol/L injectable solution. Cells were cultured as monolayers in flasks using American Type Culture Collection complete growth medium in a humidified atmosphere containing 5% CO2 at 37°C. For drug treatments, cells were seeded in complete growth media 16 hours before the experiments, in order to allow attachment but not cell-doubling. Then, cells were treated with piroxicam (760 µM) and cisplatin (4.5 µg/mL) alone or in combination for 8, 24 and 48 hours. Where indicated, i.e. P24h, cells were pretreated with piroxicam for 24 hours before adding cisplatin. Controls samples were untreated.

### Cell cycle and cell viability analysis

Unsynchronized MSTO cells (10^6^) were treated with piroxicam and cisplatin alone or in combination, as described in the previous section. Cells were harvested and stained with either propidium iodide or trypan blue. Cells stained with propidium iodide (PI) were subjected to FACS analysis, after incubation for 4 hours at 4°C in hypotonic PI solution (50 µg/ml PI, 0.1% sodium citrate, 0.1% Triton X-100, and 20 µg/ml DNase-free RNase A) then analyzed on a FACScan flow cytometer (Becton Dickinson, San Jose, CA). Histograms of cell number versus logarithm integrated FL3 fluorescence were recorded for 20.000 nuclei at flow rates no greater than 50 to 100 events per second. Cells with subdiploid DNA content (sub-G_0_/G_1_ peak) were considered apoptotic cells. Cell viability was also analyzed using the trypan blue dye exclusion method. For apoptosis analysis, harvested cells were stained with Annexin V-FITC and propidium iodide according to the manufacturer's instruction (Miltenyi Biotec, Germany) and then subjected to the same analyzer. All the experiments were performed in triplicate. Data are expressed as the mean ±SD.

### GeneChip array sample preparation

Total RNA was extracted and purified using the RNeasy Midi kit (Qiagen, Valencia, CA). Biotinylated cRNA target preparation and target hybridization to HGU133A arrays, containing 22,000 probe sets for human transcripts, were performed according to Affymetrix (Santa Clara, CA) instructions. All the hybridization, washing, staining and scanning procedures were done using a Genechip Affymetrix station (FS 450, Scanner 3000) as recommended by manufacturer. The CEL file produced by microarray scanning were used for the subsequent statistical analysis.

### GeneChip array data analysis

Four prototypic situations were analyzed to generate background-normalized image data: untreated cell line, single piroxicam or cisplatin treated cell line, piroxicam plus cisplatin treated cell line. Array analyses were carried out in triplicates for each condition. Microarray quality control and statistical validation were performed using oneChannelGUI Bioconductor package (http://www.bioconductor.org/packages/2.1/bioc/html/oneChannelGUI.html) a graphical interface used to run the analysis described below [19].

The presence of hybridization/construction artifacts was evaluated with the fitPLM function. This application allowed us to eliminate from the subsequent analysis six CEL files showing an outlier raw intensity box plot.

After probe (PM) intensity distribution evaluation, probe set intensities were obtained with GCRMA [43]. The number of genes evaluated was reduced by applying an interquartile (IQR) filter (7625 probe sets with IQR≥0.25 were retained from 22283 starting probes) followed by an intensity filter (4247 probe sets with expression signal ≥100 in at least 25% of the arrays were retained) to remove the non significant probe sets (i.e. those not expressed and those not changing) [44]. To assess differential expression between single and combined treatments, we used linear model analysis. Differential gene expression was detected using an empirical Bayes method [20] together with a false discovery rate correction of the P-value [21]. Specifically we checked differential expression in the following comparisons: piroxicam vs. control 8 hours, cisplatin vs. control 8 hours, piroxicam plus cisplatin vs. control 8 hours, piroxicam vs. control 24 hours, cisplatin vs. control 24 hours, piroxicam plus cisplatin vs. control 24 hours. Differentially expressed genes were selected using a corrected p-value threshold of 0.05 and fold change threshold of |log_2_(fc)|≥1.

Ingenuity Pathway Analysis (IPA, http://www.Ingenuity.com) was used to functionally annotate genes according to biological processes and canonical pathways.

Microarrays data reported in the manuscript were described in accordance with MIAME guidelines. Microarray data were deposited on GEO database as GSE22445 series (http://www.ncbi.nlm.nih.gov/projects/geo/).

### Quantitative Real-Time PCR analysis

Total RNA (2 µg) from each sample was converted to cDNA using High- Capacity cDNA Reverse transcription kit (Applied Biosystem, Foster City, CA) under conditions described by the supplier. Gene specific primers for the selected genes (*BIRC5*: Forward 5′ GGATCACGAGAGAGGAACATAA, Reverse 5′ TCCGCAGTTTCCTCAAATTCTT; BUB1B: Forward 5′ TCAATTGGGTTCTAAGCTGGTCTA, Reverse 5′ TCGTACACCTGGGCAAAGG; *CCNB1*: Forward: 5′GATCGGTTCATGCAGAATAATTGT 3′, Reverse 5′ CATGGCAGTGACACCAACCA 3′; *CDKN1A*: Forward 5′ CATGACAGATTTCTACCACTCCAAA, Reverse 5′ RTCCTGTGGGCGGATTAGGT; *CDKN3*: Forward: 5′ GGCAATACAGACCATCAAGCAA 3′, Reverse 5′ TGATGATAGATGTGCAGCTAATTTGT 3′; *DLG7*: Forward 5′ CGGTCCTCAGAATACGAAAAGTG, Reverse 5′ TCTATGCTGCTCCTGCTTTCAG; *FOXM1*: Forward: 5′ TGCCCGAGCACTTGGAAT 3′, Reverse 5′ CGGCGGAGCTCTGGATT 3′; *LGALS3BP*: Forward 5′ CCTTCGGGCAAGGATCAGGCCCCATCATG 3′, Reverse 5′ ACTTGCAGTCGGCCAGTGA 3′; *MAD2L1*: Forward 5′ GGGAGCGCCGAAATCG 3′, Reverse: 5′ CACGCTGATATAAAATGCTGTTGA 3′; *RACGAP*: Forward, 5′ TCCTCATGATTCACTTGCAGAGA 3′, Reverse 5′ CCAGATTGGCAACATCCATTT 3′) were designed using Primer Express 2.0 software (Applied Biosystem). GAPDH was used as internal control. Quantitative PCRs were done on an ABI PRISM 7900HT Sequence Detection System (Applied Biosystems). The entire procedure for qRT-PCR analysis - primer design, reactions, amplicon specificity and determination of gene target expression levels - was performed as previously described [23].

Relative gene expressions were calculated by relative quantification approach [45], using control samples as calibrator. Target genes were accepted as differential expressed when was ΔΔC_t_ |>1| - corresponding to 2-fold change in transcript abundance. The standard deviation was calculated for samples within each group.

### p21 sequence analysis

Primers were designed on the basis of the Ensembl Genome database sequence for Human CDKN1A. A total of 2 pairs of primers covering the two coding exons, including intron/exon junctions, were used: p21up1 Forward: 5′ CTGAGGTGACACAGCAAAGC 3′, Reverse: 5′ CAGGACCAGACAGGTCAGC 3′; p21up2 Forward: 5′ CCCAGGGAAGGGTGTCCT 3′, Reverse: 5′ CGGGAGAGAGGAAAAGGAGA 3′.

Genomic DNA from MSTO-211H cells was isolated as described by Sambrook and Russel [46]. The PCR-amplified sequences were aligned using the EMBOSS Pairwise Alignment Algorithms (http://www.ebi.ac.uk/Tools/emboss/align/). Comparison was made using as reference the CDKN1A genomic sequence from the Ensembl database (www.ensembl.org).

### Transient siRNA

Transient siRNA transfections were performed with SignalSilence p21 Waf1/Cip1 siRNA Kit (Cell Signaling Technology, Danvers, MA), according to the manufacturer's instructions with 50 nM p21 siRNA or control siRNA and Interferin (Polyplus-transfection, New York, NY) as transfection reagent. For each sample 100,000 cells/ml were plated in complete medium containing 10% FCS a day before transfection. 24 hours after transfection drug treatments were done for additional 24 hours.

### Protein extraction and Western blot analysis

Proteins gel electrophoresis, transfer and visualization were performed using standard techniques. Briefly, MSTO cells were lysed at 4°C for 1 hour in RIPA lysis buffer (150 mM NaCl, 1%NP-40, 50 mM Tris pH 8.0, 0.5% Na-deoxycholate, 0.1% SDS) supplemented with a protease inhibitor cocktail (Sigma-Aldrich, St. Louis, MO), followed by centrifugation at 14,000 g for 15′ at 4°C to separate cell debris from protein. Cytoplasmic and nuclear extracts were prepared using a nuclear extract kit (Active Motif, Carlsbad, CA) following the manufacturer's instructions.

Proteins (60 µg) were resolved on 10% SDS-PAGE gels, transferred to nitrocellulose membrane and incubated overnight at 4°C with p21, p53, CDKN3 or actin (Santa Cruz Biotechnology, Santa Cruz, CA) monoclonal antibodies. Cross contamination of nuclear and cytoplasmic fractions was excluded using RCC1 (Santa Cruz) or alpha tubulin (Abcam, Cambridge, UK) antibodies respectively. Actin was used to normalize the sample loading. Proteins were visualized with peroxidase-conjugated protein A (200 ng/ml), and ECL Plus detection reagents (Amersham, GE Healthcare Bio-Sciences Corp., Piscataway, NJ).

Electrophoretic band quantification was performed using ImageJ software (http://rsbweb.nih.gov/ij/). Statistical analysis was performed using GraphPad Prism 5.0® statistical software (GraphPad Software Inc., La Jolla, CA). Paired t test was used for comparison of two paired groups. Multiple comparisons were performed by the repeated measures ANOVA test with the Bonferroni correction for multiple.

## Supporting Information

Figure S1 **CDKN3 expression is associated to p21.** mRNA and protein levels were measured after p21 silencing. A, Real-Time PCR analysis of CDKN3 in MSTO-211H cells shows an increased expression in absence of p21. B, Western blot analysis and relative expression level of CDKN3 protein levels after p21 siRNA transient experiments. Cells transfected with control (-) or p21 siRNA were harvested at 24 hours after transfection. Total proteins were incubated with CDKN3 antibody or p21 antibody. Actin was used as loading control. Histograms of relative expression level refer to CDKN3 normalized expression and derived by the analysis of three independent experiments. Statistical analysis was done as indicated in Material and Methods.
(TIF)

Table S1 **Differentially expressed probe sets after 24 hours with piroxicam or piroxicam/cisplatin treatment.**
(XLS)

## References

[1] Robinson BW, Musk AW, Lake RA (2005). Malignant mesothelioma.. Lancet.

[2] Treasure T (2004). The learning curve.. Bmj.

[3] Crispi S, Cardillo I, Spugnini EP, Citro G, Menegozzo S (2010). Biological agents involved in malignant mesothelioma: relevance as biomarkers or therapeutic targets.. Curr Cancer Drug Targets.

[4] Pistolesi M, Rusthoven J (2004). Malignant pleural mesothelioma: update, current management, and newer therapeutic strategies.. Chest.

[5] Caraglia M, Marra M, Viscomi C, D'Alessandro AM, Budillon A (2007). The farnesyltransferase inhibitor R115777 (ZARNESTRA) enhances the pro-apoptotic activity of interferon-alpha through the inhibition of multiple survival pathways.. Int J Cancer.

[6] Fischgrabe J, Wulfing P (2008). Targeted therapies in breast cancer: established drugs and recent developments.. Curr Clin Pharmacol.

[7] Ishitsuka K, Hideshima T, Neri P, Vallet S, Shiraishi N (2008). p38 mitogen-activated protein kinase inhibitor LY2228820 enhances bortezomib-induced cytotoxicity and inhibits osteoclastogenesis in multiple myeloma; therapeutic implications.. Br J Haematol.

[8] Gordon GJ, Mani M, Maulik G, Mukhopadhyay L, Yeap BY (2008). Preclinical studies of the proteasome inhibitor bortezomib in malignant pleural mesothelioma.. Cancer Chemother Pharmacol.

[9] Vogelzang NJ, Rusthoven JJ, Symanowski J, Denham C, Kaukel E (2003). Phase III study of pemetrexed in combination with cisplatin versus cisplatin alone in patients with malignant pleural mesothelioma.. J Clin Oncol.

[10] Favaretto AG, Aversa SM, Paccagnella A, Manzini Vde P, Palmisano V (2003). Gemcitabine combined with carboplatin in patients with malignant pleural mesothelioma: a multicentric phase II study.. Cancer.

[11] Spugnini EP, Cardillo I, Verdina A, Crispi S, Saviozzi S (2006). Piroxicam and cisplatin in a mouse model of peritoneal mesothelioma.. Clin Cancer Res.

[12] Dubois RN, Abramson SB, Crofford L, Gupta RA, Simon LS (1998). Cyclooxygenase in biology and disease.. Faseb J.

[13] Leaper DJ, French B, Bennett A (1980). Reduction by flurbiprofen of primary tumor growth and local metastasis formation in mice.. Adv Prostaglandin Thromboxane Res.

[14] Altorki NK, Keresztes RS, Port JL, Libby DM, Korst RJ (2003). Celecoxib, a selective cyclo-oxygenase-2 inhibitor, enhances the response to preoperative paclitaxel and carboplatin in early-stage non-small-cell lung cancer.. J Clin Oncol.

[15] Pruthi RS, Derksen JE, Moore D (2004). A pilot study of use of the cyclooxygenase-2 inhibitor celecoxib in recurrent prostate cancer after definitive radiation therapy or radical prostatectomy.. BJU Int.

[16] Marrogi A, Pass HI, Khan M, Metheny-Barlow LJ, Harris CC (2000). Human mesothelioma samples overexpress both cyclooxygenase-2 (COX-2) and inducible nitric oxide synthase (NOS2): in vitro antiproliferative effects of a COX-2 inhibitor.. Cancer Res.

[17] Catalano A, Graciotti L, Rinaldi L, Raffaelli G, Rodilossi S (2004). Preclinical evaluation of the nonsteroidal anti-inflammatory agent celecoxib on malignant mesothelioma chemoprevention.. Int J Cancer.

[18] Spugnini EP, Crispi S, Scarabello A, Caruso G, Citro G (2008). Piroxicam and intracavitary platinum-based chemotherapy for the treatment of advanced mesothelioma in pets: preliminary observations.. J Exp Clin Cancer Res.

[19] Sanges R, Cordero F, Calogero RA (2007). oneChannelGUI: a graphical interface to Bioconductor tools, designed for life scientists who are not familiar with R language.. Bioinformatics.

[20] Smyth GK (2004). Linear models and empirical bayes methods for assessing differential expression in microarray experiments.. Stat Appl Genet Mol Biol.

[21] Wettenhall JM, Simpson KM, Satterley K, Smyth GK (2006). affylmGUI: a graphical user interface for linear modeling of single channel microarray data.. Bioinformatics.

[22] Baldi A, De Luca A, Morini M, Battista T, Felsani A (2002). The HtrA1 serine protease is down-regulated during human melanoma progression and represses growth of metastatic melanoma cells.. Oncogene.

[23] Crispi S, Calogero RA, Santini M, Mellone P, Vincenzi B (2009). Global gene expression profiling of human pleural mesotheliomas: identification of matrix metalloproteinase 14 (MMP-14) as potential tumour target.. PLoS One.

[24] el-Deiry WS, Tokino T, Waldman T, Oliner JD, Velculescu VE (1995). Topological control of p21WAF1/CIP1 expression in normal and neoplastic tissues.. Cancer Res.

[25] Sedletska Y, Giraud-Panis MJ, Malinge JM (2005). Cisplatin is a DNA-damaging antitumour compound triggering multifactorial biochemical responses in cancer cells: importance of apoptotic pathways.. Curr Med Chem Anticancer Agents.

[26] Verdina A, Cardillo I, Nebbioso A, Galati R, Menegozzo S (2008). Molecular analysis of the effects of Piroxicam and Cisplatin on mesothelioma cells growth and viability.. J Transl Med.

[27] Abbas T, Dutta A (2009). p21 in cancer: intricate networks and multiple activities.. Nat Rev Cancer.

[28] Ginsberg D (2002). E2F1 pathways to apoptosis.. FEBS Lett.

[29] Ambrosini G, Adida C, Altieri DC (1997). A novel anti-apoptosis gene, survivin, expressed in cancer and lymphoma.. Nat Med.

[30] Altieri DC (2008). Survivin, cancer networks and pathway-directed drug discovery.. Nat Rev Cancer.

[31] Kops GJ, Foltz DR, Cleveland DW (2004). Lethality to human cancer cells through massive chromosome loss by inhibition of the mitotic checkpoint.. Proc Natl Acad Sci U S A.

[32] Chang BD, Broude EV, Fang J, Kalinichenko TV, Abdryashitov R (2000). p21Waf1/Cip1/Sdi1-induced growth arrest is associated with depletion of mitosis-control proteins and leads to abnormal mitosis and endoreduplication in recovering cells.. Oncogene.

[33] Liu FT, Rabinovich GA (2005). Galectins as modulators of tumour progression.. Nat Rev Cancer.

[34] Hirose K, Kawashima T, Iwamoto I, Nosaka T, Kitamura T (2001). MgcRacGAP is involved in cytokinesis through associating with mitotic spindle and midbody.. J Biol Chem.

[35] Sillje HH, Nagel S, Korner R, Nigg EA (2006). HURP is a Ran-importin beta-regulated protein that stabilizes kinetochore microtubules in the vicinity of chromosomes.. Curr Biol.

[36] Tsou AP, Yang CW, Huang CY, Yu RC, Lee YC (2003). Identification of a novel cell cycle regulated gene, HURP, overexpressed in human hepatocellular carcinoma.. Oncogene.

[37] Laoukili J, Stahl M, Medema RH (2007). FoxM1: at the crossroads of ageing and cancer.. Biochim Biophys Acta.

[38] Costa RH (2005). FoxM1 dances with mitosis.. Nat Cell Biol.

[39] Okamoto K, Kitabayashi I, Taya Y (2006). KAP1 dictates p53 response induced by chemotherapeutic agents via Mdm2 interaction.. Biochem Biophys Res Commun.

[40] Oltersdorf T, Elmore SW, Shoemaker AR, Armstrong RC, Augeri DJ (2005). An inhibitor of Bcl-2 family proteins induces regression of solid tumours.. Nature.

[41] Gartel AL, Tyner AL (2002). The role of the cyclin-dependent kinase inhibitor p21 in apoptosis.. Mol Cancer Ther.

[42] Baldi A, Santini D, Vasaturo F, Santini M, Vicidomini G (2004). Prognostic significance of cyclooxygenase-2 (COX-2) and expression of cell cycle inhibitors p21 and p27 in human pleural malignant mesothelioma.. Thorax.

[43] Wu Z, Irizarry RA.  Stochastic models inspired by hybridization theory for short oligonucleotide arrays,.

[44] von Heydebreck A, Huber W, Gentleman RC (2004). Differential expression of the Bioconductor Project..

[45] Pfaffl MW (2001). A new mathematical model for relative quantification in real-time RT-PCR.. Nucleic Acids Res.

[46] Sambrook J, Russell D (2001).

[47] Roe OD, Anderssen E, Sandeck H, Christensen T, Larsson E (2010). Malignant pleural mesothelioma: genome-wide expression patterns reflecting general resistance mechanisms and a proposal of novel targets.. Lung Cancer.

[48] Zaffaroni N, Costa A, Pennati M, De Marco C, Affini E (2007). Survivin is highly expressed and promotes cell survival in malignant peritoneal mesothelioma.. Cell Oncol.

[49] Kim KW, Mutter RW, Willey CD, Subhawong TK, Shinohara ET (2007). Inhibition of survivin and aurora B kinase sensitizes mesothelioma cells by enhancing mitotic arrests.. Int J Radiat Oncol Biol Phys.

[50] Romagnoli S, Fasoli E, Vaira V, Falleni M, Pellegrini C (2009). Identification of potential therapeutic targets in malignant mesothelioma using cell-cycle gene expression analysis.. Am J Pathol.

[51] Stoppoloni D, Canino C, Cardillo I, Verdina A, Baldi A Synergistic effect of gefitinib and rofecoxib in mesothelioma cells.. Mol Cancer.

