# Supplementary figures and images for: Apoptosis Induced by Piroxicam plus Cisplatin Combined Treatment Is Triggered by p21 in Mesothelioma
Source: PLoS One. 2011 Aug 17;6(8):e23569. doi: 10.1371/journal.pone.0023569 (PMC3157425; doi:10.1371/journal.pone.0023569)

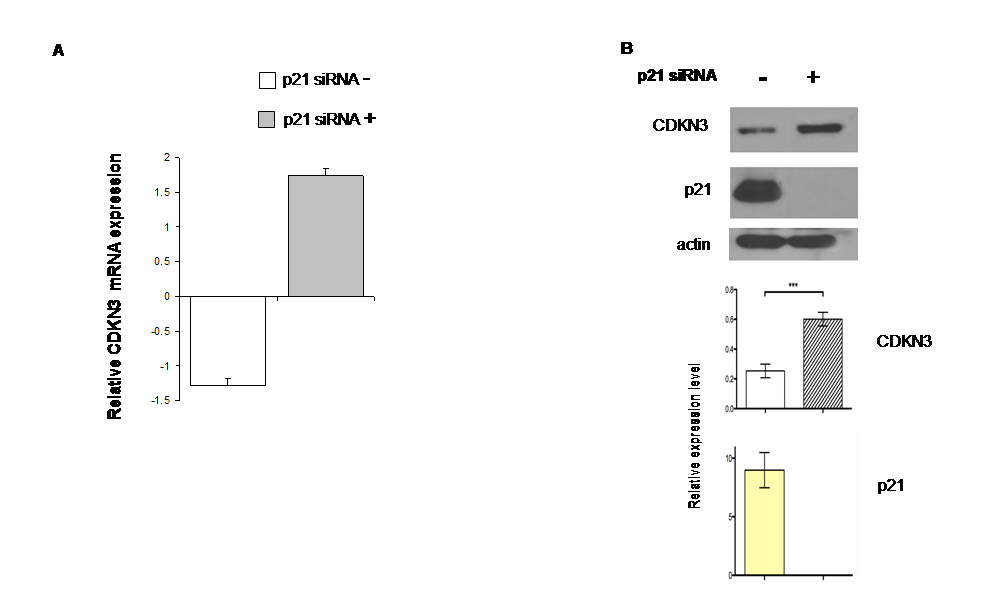

Supplement: Figure S1 — CDKN3 expression is associated to p21. mRNA and protein levels were measured after p21 silencing. A, Real-Time PCR analysis of CDKN3 in MSTO-211H cells shows an increased expression in absence of p21. B, Western blot analysis and relative expression level of CDKN3 protein levels after p21 siRNA transient experiments. Cells transfected with control (-) or p21 siRNA were harvested at 24 hours after transfection. Total proteins were incubated with CDKN3 antibody or p21 antibody. Actin was used as loading control. Histograms of relative expression level refer to CDKN3 normalized expression and derived by the analysis of three independent experiments. Statistical analysis was done as indicated in Material and Methods. (TIF) [file pone.0023569.s001.tif]
